# Newly discovered circRNAs encoding proteins: recent progress

**DOI:** 10.3389/fgene.2023.1264606

**Published:** 2023-09-27

**Authors:** Xiaotong Shi, Shiyu Liao, Zhiguo Bi, Jianguo Liu, Hua Li, Chunyang Feng

**Affiliations:** ^1^ Department of Obstetrics and Gynecology, Beijing Chao-yang Hospital of Capital Medical University, Beijing, China; ^2^ Department of Orthopedics, The First Hospital of Jilin University, Changchun, China

**Keywords:** circular RNA, encoded protein, osteosarcoma pathology, disease progression, regulatory mechanism

## Abstract

Circular RNA (circRNA) is a special class of noncoding RNA molecules and the latest research hotspot in the field of RNA. CircRNA molecules have a closed loop structure, which is not affected by RNA exonuclease and has the characteristics of more stable expression. Previous studies have shown that circRNA molecules are rich in microRNA (miRNA) binding sites and act as miRNA sponges in cells. By interacting with miRNAs associated with tumors and other diseases, circRNAs play an important regulatory role. However, circRNAs have recently been found to have small open reading frames that enable them to encode peptides/proteins. These proteins have been reported to play an important role in the mechanism of regulation of a variety of diseases and have great potential in the diagnosis and treatment of diseases. In this review, we summarize the mechanism of action of the newly discovered circRNA-coding proteins since 2022 and briefly describe their research process. In addition, we also discuss the prediction model of the functional sites and encoded proteins of circRNAs, which provides a potential idea for future research on circRNAs.

## 1 Introduction

Scientists first discovered circular RNA (circRNA) in plant viruses, yeast mitochondrial RNA and hepatitis B virus (Sanger et al., 1976; Arnberg et al., 1980; Kos et al., 1986). Different from the production of traditional linear RNA, circRNA is a new type of RNA molecule produced by back-splicing of pre-mRNA. CircRNAs have neither a 3′poly(a) tail nor a 5′cap structure and are more stable and resistant to ribonuclease degradation than linear RNAs. Initially, circRNAs were thought to be useless RNAs resulting from incorrect splicing, which could not be detected in next-generation sequencing due to the lack of a 3′poly(A) tail, thus largely hindering their identification. Due to the rapid development of high-throughput sequencing technology and bioinformatics analysis in recent years, many circRNAs have been identified, and their functions have been gradually discovered.

CircRNAs have many functions in cells (Ashwal-Fluss et al., 2014; Ng et al., 2018): 1). As a sponge for miRNA, circRNA can bind to miRNA, thereby indirectly regulating gene expression and playing the role of competing endogenous RNA. 2). CircRNAs can interact with proteins to regulate cell cycle progression by binding to cell cycle-related proteins (Du et al., 2016). 3). CircRNAs play a transcriptional regulatory role by binding to RNA polymerase II. 4). CircRNAs containing open reading frames (ORFs) and in-frame stop codons are translated into proteins in splicing-dependent and cap-independent ways. 5). Other functions include regulating intercellular signaling pathways, affecting cell differentiation, and forming pseudogenes (Memczak et al., 2013; Dong R. et al., 2016; Liu et al., 2017).

Previously, the view was usually that circRNA was an endogenous noncoding RNA (Wu et al., 2023). However, new studies have shown that circRNAs can encode proteins, many of which have been shown to play key roles in cancer pathogenesis (Legnini et al., 2017; Pamudurti et al., 2017; Zhang et al., 2018a; Zhang et al., 2018b; Yang et al., 2018; Li et al., 2021). In addition, circRNAs also have regulatory functions under other physiological and pathological conditions: they are involved in the pathogenesis of amyotrophic lateral sclerosis and frontotemporal dementia (Wang S. et al., 2021); they provide a constant supply of protein for later spermatogenesis and normal sperm function (Tang et al., 2020); they play a role in the proliferation of myoblasts (Legnini et al., 2017); and they may act as regulators of cardiovascular disease (van Heesch et al., 2019).

The results of the current studies show that cap-dependent translation of circRNAs encoding proteins is typically initiated by internal ribosome entry sites (IRES) or N6 methyladenosine (m6A)-induced ribosome engagement sites (MIRES). Lacking a 5′cap structure, circRNA follows a cap-independent translation mechanism and is dependent on IRES or MIRES to bind to the initiation factor eIF4G2 complex and anchor the 43S complex for protein translation (Godet et al., 2019; Kwan and Thompson, 2019). IRES are RNA elements that facilitate the recruitment of 40S ribosomal subunits to the internal region of mRNA to initiate translation and participate in translation initiation through a 5′-cap-independent mechanism. Studies have also revealed that under stress conditions, IRES-mediated cap-independent translation can serve as an alternative mechanism for linear mRNA production of proteins in eukaryotic cells. m6A, the methylation of adenosine base sixth nitrogen in eukaryotic RNA, is the most prevalent, abundant, and dynamically reversible accidental transcriptomic modification in mammals. Many translatable endogenous circRNAs carry m6A modification sites, which not only regulate the expression, distribution and function of circRNAs but also regulate the translation of circRNAs. The translation of circRNA is driven by MIRES as IRES-like components (Di Timoteo et al., 2020; Wang X. et al., 2021). CircRNAs are efficiently translated via 19‐nucleotide short consensus sequences (RRm6ACH) carrying m6A sites (Huang et al., 2021). The m6A-binding protein YTHDF3 recognizes m6A and recruits eIF4G2 to the m6A site and then recognizes the IRES and initiates the assembly of the eIF4 complex, recruits ribosomes and initiates translation.

## 2 New circRNA-encoded proteins regulating tumor progression

### 2.1 CircRNA-encoded proteins inhibiting tumor progression

Song et al. (2023) found that circZKSaa, a protein encoded by circZKSCAN1, inhibited hepatocellular carcinoma (HCC) progression. In their study, it was first confirmed that circZKSCAN1 encoded the circZKSaa protein by mass spectrometry, polysome fractionation assays, dual-luciferase reporter assays and other tests. They noticed that circZKSCAN1 has two internal ribosome entry sites (IRES). Then, two IRSE-site sequences between RLuc and Luc were cloned, and the results showed that both IRES sequences exhibited significantly higher Luc/Rluc activities than the empty vector, which indicated that both IRSE-1 and IRSE-2 could induce ribosome entry. They found that cell phenotypes showed suppression of HCC in both circZKSCAN1- and circZKSaa-overexpressing groups. In addition, when circZKSaa and circZKSCAN1 were overexpressed, HCC cells were significantly arrested in G0/S phase. TUNEL staining and Annexin V/PI staining showed that circZKSaa could promote the apoptosis of HCC cells. *In vivo*, the tumor volume was smaller in the circZKSaa overexpression group. Ultimately, they found that overexpression of circZKSaa promoted the interaction of FBXW7 with mammalian target of rapamycin (mTOR) and promoted the ubiquitination of mTOR, thereby inhibiting the PI3K/AKT/mTOR pathway.

Song et al. (2022) found that the circHEATR5B-encoded protein HEATR5B-881aa can inhibit aerobic glycolysis in glioblastoma multiforme (GBM). At the beginning of their study, they found that overexpression of ZVRB inhibited GBM cell proliferation and glycolysis by promoting circHEATR5B formation. To further explore the function of circHEATR5B, QT-PCR results showed that most circHEATR5B was present in the cytoplasm. A database search suggested that circHEATR5B had an open reading frame (ORF) and two internal ribosome entry sites (IRESs). The luciferase assay results showed that IRES-1 has significantly higher activity than IRES-2. The circHEATR5B-FLAG plasmid was constructed to detect the coding ability of the ORF. HEATR5B-881aa encoded by circHEATR5B was further confirmed. The expression level of HEATR5B-881aa was found to be positively correlated with the amount of circHEATR5B in GBM cells. Their study demonstrated that HEATR5B-881 reduced Jumonji C-domain-containing 5 (JMJD5) stability by phosphorylating S361. Subsequent experiments showed that JMJD5 knockdown increased pyruvate kinase M2 (PKM2) enzymatic activity and inhibited glycolysis and proliferation in GBM cells. Finally, they constructed a nude mouse xenograft model and found that overexpression of ZCRB1, circHEATR5B, and heat5b-881aa inhibited GBM growth *in vivo* and prolonged the survival time of nude mice.

Liu et al. (2023) found that the circular RNA MTHFD2L encodes the CM-248aa protein and inhibits gastric cancer progression. First, they screened DEcircRNAs with coding potential by prediction probability, ORF, IRES sequence, and ribosome analysis in three databases. They next screened circMTHFD2L, which was significantly downregulated among these circRNAs, and demonstrated its endogenous presence by gel electrophoresis. According to the predicted coding sequence of the database, the rabbit antibody of the coding protein was prepared, and the expression level of the protein was detected in cells. Finally, coimmunoprecipitation and mass spectrometry were used to verify the existence of the endogenous protein and its consistency with the predicted sequence. The inhibitory effect of CM-248aa on the proliferation and invasion of gastric cancer cells was verified by CCK-8, invasion and migration assays *in vitro*. Rescue experiments confirmed that the circular RNA MTHFD2L played a role in inhibiting gastric cancer by coding the CM-248aa protein. Mechanistically, they found that CM-248aa competitively targeted the SET nuclear oncogene (SET) and promoted the dephosphorylation of AKT, extracellular signal-regulated kinase, and P65.

Hu et al. (2022) found that the GSPT1-238aa protein encoded by circGSPT1 could also inhibit gastric cancer progression. First, they identified a novel circular RNA, circ-16168, and found that its expression was significantly reduced in GC cells compared to normal cells. They identified two IRES sequences, and the activities that mediates translation of the IRES was confirmed by luciferase assays. The results of qRT‒PCR and FISH assays showed that most of this circular RNA was located in the cytoplasm. Further analysis of the relationship between circGSPT1 and the clinicopathological index revealed that circGSPT1 expression was significantly decreased in patients with lymphatic metastasis. Therefore, the authors concluded that circRNAs were related to the invasion and metastasis of gastric cancer. Next, the IRES of circGSPT1 as well as the ORF were predicted. A FLAG-tagged plasmid was constructed to confirm that the novel protein is indeed encoded by circGSPT1. Ultimately, their study showed that the novel protein inhibits gastric cancer progression by regulating autophagy in gastric cancer cells through the PI3K/Akt/mTOR signaling pathway.

CircFBXW7 (hsa_circ_0001451) has been reported to play a tumor suppressor role in gliomas by encoding a novel protein (Yang et al., 2018). In the study by Ye et al. (2019), the downregulation of circFBXW7 in a triple-negative breast cancer (TNBC) cell line was verified by qRT‒PCR, and it was found that low expression of circFBXW7 was associated with poor clinical outcomes. They performed cell proliferation, clonal formation, Transwell, wound healing, and xenotransplantation experiments in mice, confirming that overexpression of circFBXW7 significantly inhibited cell proliferation, migration, and tumor growth. In addition, the FBXW7-185AA protein encoded by circFBXW7 inhibited the proliferation and migration of TNBC cells by increasing the abundance of FBXW7 and inducing c-Myc degradation. Therefore, circFBXW7 may serve as a therapeutic target and prognostic biomarker for TNBC.

In a study by Wang T. et al. (2021), it was reported that circASK1 (hsa_circ_0007798) was significantly downregulated in gefitinib-resistant cells and enhanced the sensitivity of EGFR-mutant lung adenocarcinoma (LUAD) cells to gefitinib. They identified a novel protein encoded by circASK1, ASK1-272A.a, which is essential for ASK1/JNK/p38 signaling activation and mediates the chemosensitivity induction of circASK1 in LAUD. One IRES in circASK1 was identified, and its activity that mediates translation was confirmed by dual-luciferase assay. This novel isomer competes with ASK1 and binds to Akt1 to antagonize Akt1-induced phosphorylation and inactivation of ASK1, thereby activating ASK1-induced apoptosis and alleviating gefitinib resistance. Clinical data and *in vivo* models further confirm the inhibitory effect of circASK1 and its encoded protein on gefitinib resistance, providing a novel therapeutic target for overcoming gefitinib resistance in LUAD patients.

### 2.2 CircRNA-encoded proteins promote tumor progression

Li et al. (2023a) found that circ-E-cad-encoded c-E-cad could promote the proliferation and invasion of gastric cancer cells. They found that circ-e-cad was localized in the cytoplasm and that the relative expression of circ-e-cad was significantly increased in GC cell lines. Therefore, they concluded that circ-e-Cad may be a useful tumor marker for GC. Next, using the prepared antibodies and immunoblotting, they found that the expression of circ-E-cad-encoded c-E-cad protein was also significantly increased in gastric cancer cells. The results of the CCK-8 assay, CFA assay, EdU assay and Transwell migration assays demonstrated that C-E-cad promoted gastric cancer cell proliferation and invasion. Subsequent rescue experiments demonstrated that the C-E-cad protein, but not cyclic RNAcE-cad, was responsible. Finally, their study demonstrated that C-E-cad promoted GC cell proliferation and epithelial-mesenchymal transition via the Tgf-β/Smad pathway.

Duan et al. (2022) identified a novel protein encoded by circMAP3K4 and demonstrated that this protein prevented cisplatin-induced apoptosis in HCC cells. First, they identified circMAP3K4 with translational potential in the CircBank database of HCC cells. After constructing a plasmid containing circMAP3K4-ORF, they found that the circMAP3K4-ORF group encoded a special polypeptide with a mass of approximately 63 kDa. Next, they demonstrated that circMAP3K4 uses m6A modification for translation rather than IRES. In subsequent *in vivo* and *in vitro* experiments, circMAP3K4-455aa was shown to promote the development of liver cancer and prevent the apoptosis of liver cancer cells. Ultimately, their study showed that circMAP3K4-455aa could organize apoptosis in HCC cells by inhibiting the cleavage and nuclear distribution of AIF.

Tang et al. (2022) identified circHNRNPU as encoding a novel protein that promotes multiple myeloma progression. First, they found that the circRNA was highly expressed in immunoglobulin D multiple myeloma (IgD MM, the most severe subtype of myeloma). Then, they confirmed the presence of circHNRNPU by trypsin digestion assay and Sanger sequencing. They found the open reading frame (ORF) of circHNRNPU and discovered the ribosomal entry site (IRES) sequence. The new protein circHNRNPU_603aa and its specific fragment were successfully identified by HNRNPU antibody and mass spectrometry, respectively, which verified the coding ability of endogenous circHNRNPU. The following MTT test, colony formation assay, flow cytometry and MM xenograft mouse model showed that the protein circHNRNPU_603aa promoted the progression of myeloma. Finally, they found that circHNRNPU_603aa includes the RNA-binding RGG-box region, which regulates SKP2 exon skipping.

Xiong et al. (2023) found that circINSIG1 encodes a novel protein that promotes colorectal cancer progression. They found that the expression of hypoxia-associated circINSIG1 was significantly upregulated in colorectal cancer. Subsequently, they verified the back-splicing junction of circINSIG1 by Sanger sequencing. The half-life and trypsin digestion results confirmed the stability of circINSIG1. Nuclear mass separation analysis and fluorescence *in situ* hybridization (FISH) analysis showed that circINSIG1 was enriched in the cytoplasm of CRC cells. They next found that the sequence of circINSIG1 contains an IRES of ORFcircINSIG1-121 with the potential to encode 121 amino acids. The activity that mediates translation of the IRES was confirmed by dual-luciferase assay. The circINSIG1-flag vector was constructed, and the expression of circIN-SIG1-121 was detected. In addition, the circIN-SIG1-121 antibody confirmed the endogenous presence of circINSIG1-121. The specific peptide fragment of the circINSIG1-121 protein was further detected by mass spectrometry and SDS‒PAGE. Finally, they found that circINSIG1-121 promoted the ubiquitination of INSIG1, a regulator of cholesterol metabolism, by recruiting the CUL5-ASB6 complex, thereby inducing cholesterol biosynthesis, which promoted CRC proliferation and metastasis.

In addition, the proteins encoded by circRNAs can also contribute to cancer progression by enhancing resistance to anticancer drugs. Oxaliplatin is commonly used in chemotherapy for colorectal cancer (CRC) after surgical resection. Pan et al. (2022) found that circATG4B was increased in exosomes secreted by oxaliplatin-resistant CRC cells and induced oxaliplatin resistance by promoting autophagy. Further *in vivo* and *in vitro* studies indicate that the effect of circATG4B is attributed to its potential to encode a novel protein, circATG4B-222aa. One IRES in circATG4B was identified, and its activity that mediates translation was confirmed by dual-luciferase assay. CircATG4B-222aa competitively interacts with TMED10 and prevents TMED10 from binding to ATG4B, which leads to increased autophagy and the subsequent induction of chemoresistance. This study suggests that exosomal circATG4B is involved in reducing the chemosensitivity of CRC cells and provides a new basis for a potential therapeutic target for oxaliplatin resistance in CRC.

Li et al. (2022) identified a functional RNA, hsa_circ_0000384 (circMRPS35), that is highly expressed in hepatocellular carcinoma (HCC). Knockdown of circMRPS35 expression inhibited HCC cell proliferation, migration, invasion, colony formation and the cell cycle *in vitro* and tumor growth *in vivo*. In addition, they detected a peptide encoded by circMRPS35 (circMRPS35-168aa) that was significantly induced by chemotherapy drugs and promoted cisplatin resistance in HCC. In their study, two internal ribosome entry sites (IRESs) were identified, and luciferase assay results showed that IRES-1 has significantly higher activity than IRES-2. These results suggest that circMRPS35 may be a novel mediator in HCC progression, and they raise the potential of novel biomarkers for HCC diagnosis and prognosis, as well as novel therapeutic targets for HCC patients.

**FIGURE 1:**
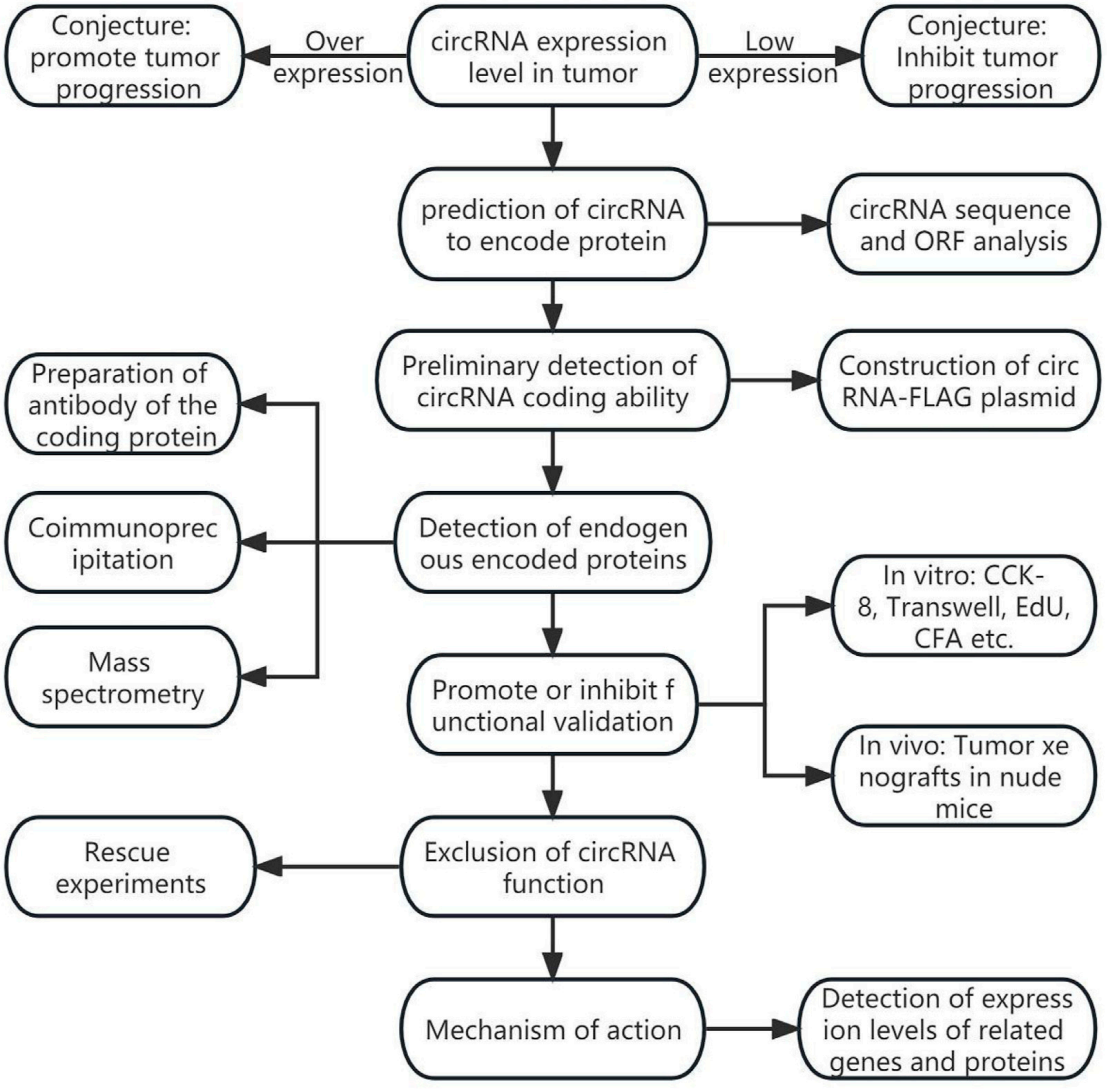
General process of tumor-associated circular RNA coding protein discovery.

The coding information of tumor-associated circRNAs is summarized in Table 1; Figure 1 shows the general discovery and research process of tumor-associated circRNA-encoded proteins.

**TABLE 1:** Proteins encoded by circRNAs regulate tumor progression.

circRNA	Manily genomic location	Genomic expression abundance in tumor tissue	Number of IRES	Whether translation in frame	Encoded protein	Main function	Mechanism	Reference
circZKSCAN1	Cytoplasm	Downregulated	2	Yes	circZKSaa	Inhibit hepatocellular carcinoma	Inhibiting PI3K/AKT/mTOR pathway	Song et al. (2023)
circHEATR5B	Nuclear	Downregulated	2	Yes	HEATR5B-881aa	Inhibit Glioblastoma multiforme	Reducing JMJD5 stability	Song et al. (2022)
circMTHFD2L	nuclear	Downregulated	1	Yes	CM-248aa	Inhibit gastric cancer	Promoting dephosphorylation of AKT	Liu et al. (2023)
circGSPT1 circFBXW7	Cytoplasm nuclear and cytoplasm	Downregulated	2	Yes	GSPT1-238aa	Inhibit gastric cancer	Regulating autophagy through PI3K/Akt/mTOR signaling pathway	Hu et al. (2022); Ye et al. (2019)
		Upregulated	—	Yes	FBXW7- 185aa	Promote gastric cancer	Regulating Tgf-β/Smad pathway	
circASK1	Cytoplasm	Downregulated	1	Yes	ASK1-272A.a	Enhance the sensitivity of EGFR-mutant lung adenocarcinoma (LUAD) cells to gefitinib	Activating ASK1-induced apoptosis and alleviating gefitinib resistance	Wang et al. (2021c); Li et al. (2023a)
circ-E-cad	Cytoplasm	Downregulated	—	Yes	C-E-cad	Inhibit Triple-Negative Breast Cancer	Inhibiting the proliferation and migration of TNBC cells by increasing the abundance of FBXW7 and inducing c-Myc degradation	
circMAP3K4	Cytoplasm	Upregulated	2	Yes	circMAP3K4-455aa	Prevent hepatocellular carcinoma apoptosis	Inhibiting cleavage and nuclear distribution of AIF	Duan et al. (2022)
circHNRNPU	Cytoplasm	Upregulated	1	Yes	circHNRNPU_603aa	Promote multiple myeloma	Regulating SKP2 exon skipping and competitive inhibition of c-Myc ubiquitin	Tang et al. (2022)
circINSIG1	Cytoplasm	Upregulated	1	Yes	circIN-SIG1-121	Promote colorectal cancer	Promoting ubiquitination of INSIG1 and inducing cholesterol biosynthesis	Xiong et al. (2023)
circATG4B	Cytoplasm	Upregulated	1	Yes	circATG4B‐222aa	Induce oxaliplatin resistance in colorectal cancer	Interacting with TMED10 and preventing TMED10 from binding to ATG4B, which leads to increased autophagy and then induction of chemoresistance	Pan et al. (2022)
circMRPS35	Cytoplasm	Upregulated	2	Yes	circMRPS35-168aa	Promote cisplatin resistance in HCC	CircMRPS35 was significantly induced by chemotherapy drugs and promoted cisplatin resistance in HCC	Li et al. (2022)

## 3 CircRNA-encoded proteins and viruses

Zhang et al. (2022) found that circRNA-vSP27 derived from *Bombyx mori* cypovirus (BmCPV) encodes the viral peptide vSP27 against viral infection. First, they identified a novel polypeptide, vSP27, from the BmCPV minus strand, identified a circRNA encoded by the BmCPV genome and verified a head and tail sequence from Sanger sequencing. The pIZT-LcR-circRNA-vSP27 vector was then prepared, and the head and tail sequences of circRNA-vSP27 could be detected in the transfected cells. The translational activity of circRNA-vSP27 was confirmed by Western blotting in pIZT-lcr-circRNA-vSP27-transfected cells. However, the expression level of vSP27 was affected by circ-siRNA but not by linear siRNA treatment, indicating that vSP27 was mainly translated by circRNA-vSP27. They then found that the expression of viral structural protein VP7 and viral structural protein genes (vp1 and vp2) was decreased in BmCPV-infected cells overexpressing circRNA-vSP27. Finally, their study showed that ROS production induced by vSP27 activated the NF-κB signaling pathway and induced the expression of antimicrobial peptides, thereby inhibiting BmCPV infection.

However, in the study by Zhang Y. et al. (2023), the peptide VSP39 encoded by circRNA-000010 could promote viral replication. They found that circRNA-000010 expression was detectable in cells 6 h after infection with *Bombyx mori* Nucleopolyhedrovirus (BmNPV) and increased with viral infection. First, they found that circRNA-000010 has a small open reading frame (sORF) and an IRES-like element. The expression of VSP39 protein was then detected by Western blotting in BmnPV-infected midgut and BmN cells and in cells transfected with the circ_000010 plasmid. In BmN cells pretransfected with circ_000010, Western blot results showed that knocking down the linear transcript did not change the expression of VSP39, while knocking down circRNA-000010 significantly reduced the expression of VSP39. The translation of VSP39 from circ_000010 was demonstrated. Finally, the VSP39 vector was prepared using the ORF of VSP39 and transfected into BmN-infected cells. The expression of the Bm59 gene and GFP and virus titer increased with increasing vector transfection dose of VSP39. This proves that VSP39 can promote viral replication.

In a study by Amaya et al. (2023), a circular RNA vaccine induced enhanced T-cell responses. First, they studied the immune response induced by circRNAs. They conjugated circRNA to the fluorescent vector AF488 and subcutaneously injected AF488-circRNA into C57BL/6 mice. Circular RNA uptake was detected by flow cytometry in monocytes, dendritic cells and several macrophage subsets in inguinal draining lymph nodes (iLNs) at 24 h postimmunization. The B-cell frequency in iLNs was significantly increased at 24 h postimmunization, and the monocyte frequency and activation were significantly increased compared with those in untreated controls. Flow cytometry results confirmed that circular RNA treatment significantly induced the expression of the proinflammatory cytokine genes IL-1β, TNFa, and IL-6, and these cytokines promoted dendritic cell differentiation and maturation. Then, they designed a circRNA-encoding chick ovalbumin (circOVA), and CARTS (degradable biological material for efficient mRNA delivery) was used for intraperitoneal delivery in mice. At 7 days after a single immunization, they observed that CART-circOVA induced robust CD8 T-cell responses in the lung, spleen, and blood. Three weeks after booster immunization, significant levels of CD8 T-cell responses were observed in the spleen and lung tissues of mice. These results suggest that circRNA-encoded antigens could induce strong T-cell responses *in vivo*.

Chen et al. (2023a) investigated ways to increase the efficiency of engineered circular RNA-encoding proteins. First, they created a modular cloning platform to enable high-throughput circRNA detection. Then, they systematically studied the basic elements that control circular RNA translation, including vector topology, 5′and 3′UTRs, IRESs and synthetic aptamers. Their studies on vector topology showed that increasing the length of the interval between translation initiation and the splicing scar was detrimental to translation and that some ribose was unaffected by the secondary structure of the splicing scar. CircRNAs with a spacer of 50 nucleotides (nt) yielded the strongest translation. Their study also showed that both 5′and 3′UTRsA and full-length viral IRES can promote strong translation. Finally, they combined the best components (upstream IRES topology, 5′PABP spacer, HBA1 3′UTR and HRV-B3 IRES with proximal loop Apt-eIF4G insertion) to test the ability of circular RNA expression and found that the ability of circular RNA to translate proteins was increased by hundreds of times.

For diseases caused by viruses, circRNA-encoded proteins can also play a regulatory role. Transmissible gastroenteritis virus (TGEV), a member of the coronavirus family, is the pathogen that causes infectious gastroenteritis and can cause mitochondrial dysfunction in host cells. Transmissible gastroenteritis virus (TGEV), a member of the coronavirus family, is the pathogen that causes infectious gastroenteritis and can cause mitochondrial dysfunction in host cells. In addition, the mitochondrial permeability transition is caused by the abnormal opening of the mitochondrial permeability transition pore (mptp) regulated by the voltage-dependent anion-selective channel protein 1 (VDAC)-cyclophilin D (CypD) complex, which is a critical step in the process of mitochondrial dysfunction. Zhao et al. (2022) found that circBIRC6-2 encodes a new protein named BIRC6-236aa. This protein inhibits TGEV-induced mptp opening in TGEV infection. Mechanistically, BIRC6-236aa interacts with VDAC1 to destroy the stability of the VDAC1-CYPD complex. These results suggest that a new protein, BIRC6-236aa, encoded by circBIRC6-2, inhibits mPTP opening and subsequent mitochondrial dysfunction by interacting with VDAC1.

## 4 New circRNA-encoded proteins and the urogenital system

According to previous reports, circRNAs are involved in the regulation of a variety of pathophysiological processes in the human body and play roles in cardiovascular system diseases, myoblast proliferation, and nervous system diseases. In the latest study, the authors found that circRNAs also play an important role in genitourinary system-related physiological processes and diseases.

CircRNA has been shown to be abundant in the mammalian testis (Dong WW. et al., 2016; Li et al., 2017) and was found to be differentially expressed in patients with nonobstructive azoospermia, suggesting a role in spermatogenesis (Ge et al., 2019). Approximately half of the circRNAs in mouse male germ cells contain open reading frames with m6A-modified start codons, suggesting their coding potential (Tang et al., 2020). Zhang S. et al. (2023) reported an endogenous circRNA (circRsrc1) that encodes a new protein. They named the protein Rsrc1-161aa and found that it was made up of 161 amino acids. Knocking out Rsrc1-161aa resulted in a significant decrease in sperm count and motility in mice due to mitochondrial energy metabolism disorder, resulting in impaired male fertility. A series of *in vitro* rescue experiments showed that circRsrc1 regulates mitochondrial function through its encoded protein Rsrc1-161aa. Further investigation of the mechanism revealed that Rsrc1-161aa interacts with the mitochondrial protein C1qbp directly, enhancing its binding activity to mitochondrial mRNA, thereby regulating mitochondrial ribosome assembly and affecting the translation of OXPHOS proteins and mitochondrial energy metabolism. Therefore, the authors concluded that the circRsrc1-encoded Rsrc1-161aa protein regulates mitochondrial ribosome assembly and translation during spermatogenesis, thereby affecting male fertility.

Ouyang et al. (2023) reported an endogenous circRNA: circ-ZNF609. It originates from the ZNF609 locus and is highly expressed in the kidney after ischemia‒reperfusion injury. Overexpression of circ-ZNF609 activated AKT3/mTOR signaling, inhibited the proliferation of HK-2 cells and induced impaired autophagic flow and apoptosis, while silencing circ-ZNF609 blocked this effect. Further investigation of the mechanism revealed that circ-ZNF609 encodes a functional protein named ZNF609-250aa, which is composed of 250 amino acids. One IRES in circ-ZNF609 was identified, and its activity that mediates translation was confirmed by dual-luciferase assay. The overexpression of circ-ZNF60 can activate AKT3/mTOR signaling and induce autophagy flux impairment and cell apoptosis in HK-2 cells *in vitro* and in AKI kidneys *in vivo*. Blockade of the AKT and mTOR signaling pathways by pharmacological inhibitors reversed the impaired autophagic flow and apoptosis induced by znf609-250a in HK-2 cells. This study demonstrated that the highly expressed circ-ZNF609-encoded ZNF609-250aa induced apoptosis and AKI by impairing autophagic flow through an AKT/MTOR-dependent mechanism. Targeting circ-ZNF609 may be a novel treatment for ischemic AKI.

Zhu et al. (2021) explored the possible role of circRNA and its encoded protein in the occurrence and development of bladder outlet obstruction (BOO). They used partial bladder outlet obstruction to establish the BOO model in rats and then used deep RNA sequencing and isobaric tags for relative and absolute quantification (iTRAQ) quantitative proteomics techniques to analyze the differentially expressed circRNAs and protein profiles, respectively. GETORF software was used to predict the open reading frame (ORF) of the new protein encoded by circRNA, which was verified by mass spectrometry in proteomics, and its expression changes were verified by quantitative RT‒PCR. A total of 3 051 circRNAs were differentially expressed in bladder tissues of BOO rats, including 1 414 upregulated circRNAs and 1 637 downregulated circRNAs. Our subsequent quantitative proteomics results showed that 85 proteins were significantly altered in the rat BOO model and were enriched in multiple biological processes and signaling pathways, such as the PPAR and Wnt pathways. Twenty-one differentially expressed proteins were predicted to be encoded by circRNAs, and the levels of circRNAs and proteins were consistent in the rat BOO model. The expression levels of circRNAs were further verified by quantitative RT‒PCR and mass spectrometry. The circRNA and protein profiles were significantly changed in the rat BOO model, and the expression of the new protein encoded by circRNA was greatly changed. These results suggested that circRNAs may play a potential new role in the occurrence and development of BOO through the signaling pathway induced by the novel protein they encode.

Nontumour-associated circRNA coding information is presented in Table 2.

**TABLE 2:** Functions of nontumour-associated proteins encoded by circRNAs.

circRNA	Manily genomic location	Genomic expression abundance in lesion tissue	IIRES	Whether translation in frame	Encoded protein	Main function	Mechanism	Reference
circRNA-vSP27	Cytoplasm	—	—	Yes	vSP27	Inhibit BmCPV infection.	Activate NF-κB signaling pathway and induce antimicrobial peptides expressiion	Zhang et al. (2022)
circRNA-000010	Cytoplasm	—	1	Yes	VSP39	Promote BmNPV replication	Upregulate Bm59 gene expression, GFP expression	Zhang et al. (2023a)
AF488- circRNA	—	—	1	Yes	CircOVA	Induce strong T-cell responses *in Vivo*	Promote dendritic cell differentiation and maturation	Amaya et al. (2023)
circBIRC6-2	Cytoplasm	—	2	Yes	BIRC6-236aa	Inhibit mPTP opening and subsequent mitochondrial dysfunction by interacting with VDAC1.	BIRC6-236aa interacts with VDAC1 to destroy the stability of VDAC1-CYPD complex.	Zhao et al. (2022)
circRsrc1	Cytoplasm	—	—	Yes	Rsrc1-161aa	Play a role in spermatogenesis	Affect translation of oxidative phosphorylation proteins and mitochondrial energy metabolism	Zhang et al. (2023b)
circ-ZNF609	Cytoplasm	Upregulated	1	Yes	ZNF609-250aa	Induce autophagy flux impairment and cell apoptosis	Impairing autophagic flow through an AKT/MTOR-dependent mechanism	Ouyang et al. (2023)
circTmeffff1	Cytoplasm	Upregulated	1	Yes	TMEFF1-339aa	Promotes Muscle Atrophy	Cyclic GMP-AMP synthase (cGAS)/stimulator of interferon genes (STING) pathway	Chen et al. (2023b)
circAβ-a	—	Upregulated	—	Yes	Aβ175	Play a role in the pathology of Alzheimer’s disease	—	Mo et al. (2020)
circNlgn	Nuclear	Upregulated	—	Yes	Nlgn173	aberrant collagen deposition, cardiac fibroblast proliferation, and reduced cardiomyocyte viability	Bind to both ING4 and C8orf44-SGK3 promoters,	Du et al. (2021)

## 5 CircRNA-encoded proteins in other systems

### 5.1 CircRNA-encoded proteins in muscle tissue

CircRNAs play an important role in the proliferation of myoblasts. Legnini et al. (2017) analyzed circRNA expression profiles during *in vitro* differentiation of murine and human myoblasts. They identified conserved circRNAs regulated during murine and human muscle differentiation and that their expression was altered in myoblasts with Duchenne muscular dystrophy (DMD). Using a dedicated knockdown strategy and subsequent high-content functional genomic screening, they identified circRNAs involved in the regulation of myogenesis. Among them, circ-ZNF609 could specifically control the proliferation of myoblasts. Circ-ZNF609 contains an open reading frame that, similar to the linear transcript, begins at the start codon and terminates at an in-frame stop codon created upon circularization. Circ-ZNF609 associates with heavy polyribosomes and is translated into proteins in both a splicing-dependent and cap-independent manner, providing an example of a protein-coding circRNA in eukaryotes.

Skeletal muscle atrophy is a common clinical feature of many acute and chronic diseases. Chen et al. (2023b) analyzed the expression profile of circRNA and showed that circRNA was involved in the pathophysiological process of muscle atrophy. CircTmeff1 was identified as a potential circRNA candidate affecting muscle atrophy. Furthermore, circTmeff1 was found to be highly expressed in multiple types of muscle atrophy *in vitro* and *in vivo*, and knockdown of circTmeff1 partially rescued the muscle mass of mice in the established atrophic environment. Mechanistically, circTmeff1 directly interacts with TAR DNA-binding protein 43 (TDP-43) and promotes TDP-43 accumulation in mitochondria, thereby triggering mitochondrial DNA (mtDNA) release into the cytoplasm. It also activates the cyclic GMP-AMP synthase (cGAS)/stimulator of interferon genes (STING) pathway. Unexpectedly, TMEFF1-339aa was identified as a novel protein encoded by circTmeff1 that mediates its proatrophic effect, and one functional IRES was identified by dual-luciferase assay.

### 5.2 CircRNA-encoded proteins in the nervous system

In the nervous system, an increasing number of studies have shown that circRNAs are involved in the regulation of related proteins in the pathogenesis of Alzheimer’s disease (Mo et al., 2020). Amyloid beta-peptide (Aβ) plays a crucial role in the pathological process of AD. In familial AD, Aβ is produced from full-length β-amyloid precursor protein (APP) through dysregulated proteolysis; however, the mechanism of Aβ biogenesis in sporadic AD remains unclear. Mo et al. (2020) identified a circular RNA containing the Aβ coding region of the APP gene, called circAβ-a. They detected this circRNA in a comparison of AD patient brains with nondemented human brains, demonstrating that circAβ-a was efficiently translated into a novel Aβ175 polypeptide (19.2 kDa) containing Aβ in both cultured cells and human brains. In addition, they found that Aβ175 is processed into the Aβ peptide, which is a hallmark of AD. This study reveals another pathway for Aβ biosynthesis, and circAβ-a and its translation products may be novel targets for AD treatment.

The C9ORF72 hexanucleotide GGGGCC repeat expansion is the most common genetic cause of amyotrophic lateral sclerosis (ALS) and frontotemporal dementia (FTD). However, it is not clear how these intronic repeats are exported and translated in the cytoplasm. Using a single-molecule imaging approach to examine the molecular identity and spatiotemporal dynamics of the repeat RNA, Wang S. et al. (2021) demonstrated that C9ORF72 is a circRNA composed of spliced introns rich in G repeats and is involved in the pathogenesis of amyotrophic lateral sclerosis and frontotemporal dementia. This circular intron serves as a translation template for the toxic dipeptide repeat protein. Interestingly, this translation is driven by repeat-related non-AUG translation. This study reveals an uncharacterized disease-causing RNA species mediated by repeat expansions and demonstrates the importance of RNA spatial localization for understanding disease etiology.

### 5.3 CircRNA-encoded proteins in the cardiovascular system

In a human heart translatome study (van Heesch et al., 2019), researchers identified hundreds of previously undetected microproteins from lncRNAs and circRNAs and validated these protein products *in vivo*. The translation of microproteins is not restricted to the heart and is prominent in the translatome of the human kidney and liver. They linked these microproteins to different cellular processes and compartments and found that many microproteins localized to mitochondria. Importantly, many microproteins are translated from lncRNAs with well-defined noncoding functions, suggesting previously unrecognized biological functions. In the study by Du et al. (2021), the underlying mechanisms of fibrotic cardiac remodeling that can lead to arrhythmias and progressive heart failure were investigated. Using RNA sequencing, they found that circular neuroligin RNA (circNlgn) was highly upregulated in myocardial tissue from patients with cardiac overload in specific congenital heart diseases. Backsplicing of the neuroconnexin gene resulted in the translation of a circular RNA-derived peptide (Nlgn173) with a 9-amino acid nuclear localization motif. Nlgn173 binds to the ING4 (growth protein inhibitor 4) and C8orf44-SGK3 (serum and glucocorticoid-induced kinase 3) promoters, resulting in abnormal collagen deposition, cardiac fibroblast proliferation, and reduced cardiomyocyte viability. In the mouse experiment, three-dimensional ultrasound imaging revealed impaired left ventricular function in circNlgn transgenic mice. In addition, circRNA-encoded peptides may also function as regulators in cardiovascular diseases, such as ischemic cardiomyopathy, drug-induced cardiomyopathy, and other cardiac pathological states.

### 5.4 Role of circRNA in the aging process

Proteins encoded by circRNAs may also play a role in regulating the aging process. Researchers (Weigelt et al., 2020) found that the accumulation of circRNAs was slowed in long-lived insulin-mutant flies. Next, they determined the *in vivo* function of circRNAs generated by the sulfate-free gene (circSfl), which was consistently upregulated in multiple long-lived insulin mutants, especially in brain and muscle. Notably, the lifespan extension of the insulin mutant was circSfl dependent, whereas overexpression of circSfl alone was sufficient to extend lifespan. In addition, circSfl is translated into a protein that shares the N-terminus with the full-length Sfl protein encoded by the host gene and may have some function. This study demonstrates that insulin signaling affects global circRNA accumulation and reveals an important role for circSfl during aging *in vivo*.

## 6 Prediction models of circRNA function and structure

Due to the large number of circular RNAs, functional and structural prediction models are essential to improve the efficiency of research. Therefore, in this section, we mainly discuss the novel structure and function prediction models of circRNAs.

In the past, circRNAs were found to be regulated by binding to related RNA-binding proteins (RBPs). Du et al. and Li et al. proposed multiview-based circRNA binding site prediction models. Duan et al. first used four different feature coding methods to extract the feature information of RNA. Then, a global union of multiple views is generated to construct the intrinsic connection between views, which is used for feature calibration of each view, highlighting important features and suppressing unimportant features. Finally, the binding sites of the circRNAs were detected using the depth features obtained by multiview fusion. In 37 ciRNA-RBP databases, the area under the curve (AUC) of this method was as high as 93.68%. The code and data sets are available from https://github.com/Xuezg/JLCRB. (Du and Xue, 2022). The advantage of the prediction model is that the proposed mixed nucleotide frequency enriches the local context information of the sequence, while the location-specific trinucleotide tendency calculates the sequence context tendency from a global perspective. These four different views can achieve complementarity between information and a more comprehensive representation of the sequence’s features. The drawback is that it is computationally intensive and may omit some view-specific features that are useful for model prediction. However, Li et al. used five feature coding schemes to construct multiviews. A global representation of each view is then generated to calibrate features and filter out redundant information. Finally, multiple views were fused to detect RNA-binding sites. The AUC of 37 database performances of the model is 93.85%, and the source code is available at https://github.com/dxqllp/ASCRB. (Li L. et al., 2023). Compared with the JLCRB model proposed by Du et al., the advantage of ASCRB is that it can screen more useful features and suppress less important features in the process of feature extraction, thus eliminating redundant information in each view.

Dal Molin et al. (2022) developed CRAFT (CircRNA Function Prediction Tool), a free computational pipeline that not only predicts circRNA sequences and molecular interactions with miRNAs and RBPs but also their coding potential. First, the software can provide the sequence of putative circRNAs, which provides a basis for predicting the coding function. Second, the software can directly predict the three main functions of circRNAs: binding miRNA, binding RBPS and coding proteins. It is worth mentioning that the software can predict the coding potential of circular RNA circular forms but not linear coding potential. Currently, circRNA functional predictions are provided by web databases that do not allow custom analyses, while self-standing circRNA prediction tools are mostly limited to predicting only one type of function, mainly focusing on the miRNA sponge activity of circRNA. CRAFT provides a comprehensive graphical visualization of the results, links to several knowledge databases, and extensive functional enrichment analysis.

In the study of circRNAs, the association between diseases and circRNAs is also very important. The emergence of prediction models for circRNA-disease associations helps to discover more important circRNAs. A computational framework for predicting potential circRNA-disease associations, RGCNCDA, was proposed in the study by Chen et al. (2022) First, the circRNA-miRNA-disease global heterogeneous network was constructed by integrating the circRNA similarity network, miRNA similarity network, disease similarity network and the interaction or association network between them. Second, low- and higher-order structural information is obtained from global heterogeneous networks. Finally, they constructed a prediction model using the R-GCN encoder and DistMult decoder to infer novel potential circRNA-disease associations. Although the current research on the relationship between circular RNA and disease has achieved a certain degree of success, many methods have not fully considered pathogenic factors and have failed to analyze the relationship between circRNA and disease at the level of biological systems, and the biological features obtained are not comprehensive enough. RGCNCDA makes full use of circRNA similarity, miRNA similarity, disease similarity and the known association information between three biological entities to build a global heterogeneous network. RGCNCDA outperformed six other state-of-the-art approaches in fivefold cross-validation experiments.

In their study, Yang and Chen (2023) proposed a method for predicting circRNAs and drug sensitivity (MNGACDA). First, they constructed a multimodal network using multiple information sources from cities and drugs. Then, the low-dimensional embeddedness of circRNAs and drugs was obtained from the multimodal network. Finally, based on the embedded representation of circRNA and drug, the inner product decoder was applied to predict the association between circRNA and drug sensitivity. A large number of experimental results based on cross-validation show that MNGACDA is superior to the other six most advanced methods and will save considerable manpower and material resources.

Table 3 summarizes information about circRNA-related prediction procedures or methods.

**TABLE 3:** CircRNA-related prediction programs and methods.

Name	Prediction functions	Data availability	Reference
JLCRB	CircRNA binding site prediction	https://github.com/Xuezg/JLCRB	Du and Xue (2022)
ASCRB	CircRNA binding site prediction	https://github.com/dxqllp/ASCRB	Li et al. (2023b)
CRAFT	CircRNA sequences	https://github.com/annadalmolin/CRAFT	Dal Molin et al. (2022)
	Interactions with miRNAs/RBPs		
	Coding potential		
RGCNCDA	Potential circRNA-disease associations	—	Chen et al. (2022)
MNGACDA	CircRNAs and drug sensitivity	https://github.com/youngbo9i/MNGACDA	Yang and Chen (2023)

## 7 Conclusion

This article summarizes the latest research results on circRNA-encoding proteins. There is increasing evidence that circRNAs have protein-coding capabilities, produce functional proteins or peptides, and participate in various biological processes. Most previous studies have focused on the role of circRNAs in the occurrence and progression of cancer. CircRNA-encoded peptides can act as either oncogenes or tumor suppressors, residing in the nucleus, cytoplasm, cell membrane, or secreted proteins. Because circRNA-encoded proteins have significant sequence overlap with their cognate mRNAs and use the same reading frame, most circRNA-encoded proteins exert their function by enhancing or attenuating the activity of the original protein encoded by the corresponding mRNA. In recent years, researchers have identified the role of circRNAs in cardiovascular system diseases, myoblast proliferation, and nervous system diseases. Recent studies have also reported that circRNAs play an important role in genitourinary system-related physiological processes and diseases. Although most studies have used Western blot and immunofluorescence assays to analyze the expression level of the target protein, a rescue assay is still needed to determine whether the protein, rather than the circRNA itself, plays a physiological or pathological role.

At present, substantial progress has been made in the regulation and function of circRNAs, but there are still many valuable directions for future research. First, most of the activities of circRNA-encoded proteins reported thus far are associated with cancer. Future studies should further explore whether these proteins encoded by circRNAs play important roles in other physiological and pathological processes, such as developmental lung diseases and cell proliferation and differentiation during embryonic development. Second, many circRNAs can either encode proteins or possibly act as sponge molecules to inhibit the action of microRNAs; which of these two functions is the primary activity of these circRNAs needs to be further investigated. Third, the physiological and pathological processes in the human body are the result of the interaction of many factors. Therefore, the correlation between the expression level of the circRNA-encoded protein and physiological or pathological processes should be fully investigated. In summary, the current research achievements on circRNAs are only the tip of the iceberg in this field. Further study of circRNA-related networks in the pathogenesis of cancer, as well as their role in other physiological and pathological processes, will promote our understanding of complex cell signaling pathways and regulatory mechanisms and will be one of the most popular research directions in the future.
